# AMPK-mTOR pathway is involved in glucose-modulated amino acid sensing and utilization in the mammary glands of lactating goats

**DOI:** 10.1186/s40104-020-0434-6

**Published:** 2020-02-14

**Authors:** Jie Cai, Diming Wang, Feng-Qi Zhao, Shulin Liang, Jianxin Liu

**Affiliations:** 1 Institute of Dairy Science, College of Animal Sciences, Zhejiang University, Hangzhou, 310058 People’s Republic of China; 2 Department of Animal and Veterinary Sciences, University of Vermont, Burlington, VT 05405 USA

**Keywords:** Amino acid profile, AMPK signaling, Glucose supply, Mammary gland, Milk protein component

## Abstract

**Background:**

The local supply of energy-yielding nutrients such as glucose seems to affect the synthesis of milk components in the mammary gland (MG). Thus, our study was conducted to investigate the effects of locally available MG glucose supply (LMGS) on amino acid (AA) sensing and utilization in the MG of lactating dairy goats. Six dosages of glucose (0, 20, 40, 60, 80, and 100 g/d) were infused into the MG through the external pudendal artery to investigate the dose-dependent changes in mammary AA uptake and utilization (Exp.1) and the changes in mRNA and protein expression of the AMPK-mTOR pathway (Expt.2).

**Results:**

In Exp.1, total milk AA concentration was highest when goats were infused with 60 g/d glucose, but lower when goats were infused with 0 and 100 g/d glucose. Increasing LMGS quadratically changed the percentages of α_S2_-casein and α-lactalbumin in milk protein, which increased with infusions from 0 to 60 g/d glucose and then decreased with infusions between 60 and 100 g/d glucose. The LMGS changed the AA availability and intramammary gland AA utilization, as reflected by the mammary AA flux indexes. In Exp.2, the mRNA expression of *LALBA* in the MG increased quadratically with increasing LMGS, with the highest expression at dose of 60 g/d glucose. A high glucose dosage (100 g/d) activated the general control nonderepressible 2 kinase, an intracellular sensor of AA status, resulting in a reduced total milk AA concentration.

**Conclusions:**

Our new findings suggest that the lactating MG in dairy goats may be affected by LMGS through regulation of the AA sensory pathway, AA utilization and protein synthesis, all being driven by the AMPK-mTOR pathway.

## Background

Local nutrition, being an important part of precision nutrition, relates to nutrient supply, uptake and utilization in a specific organ, tissue or cell [1]. The functional mammary gland (MG) serves as a lactating organ and its nutrition is of high importance. Understanding of local mammary nutrition is therefore critical for improving milk production and milk quality [2, 3]. The local supply of energy-yielding nutrients such as glucose appears to impact both female metabolism and bovine synthesis of milk components [4]. In our previous study, increased glucose supply to the MG largely affected milk glucose-related metabolites and led to acute glycolysis and oxygen radical accumulation in the goat MG at high dosages of glucose [5], indicating that glucose availability and intracellular metabolic pathways can be altered by glucose supply to the MG. However, it is unclear whether the local glucose supply may also alter the amino acid (AA) metabolism in the MG and milk AA concentration.

The mammalian target of rapamycin (mTOR) signaling pathway is known to play an important role in sensing and responding to changes in the availability of local nutrients, such as AA [6, 7]. The AA sensory function of the mTOR pathway was first demonstrated in a study of hepatocyte autophagy by Blommaart et al. [8], who showed that addition of AA inhibited valine release as a result of autophagic protein degradation and the effect was inhibited by rapamycin. Recently, the mTOR pathway has been shown to be critical for AA sensing and utilization in the lactating bovine or mouse MG [9, 10].

Milk protein synthesis can be manipulated by both protein and energy supply [11–13]. Mammary AA metabolism can be influenced by changes in the AA profile and other nutrients in the mammary arterial blood [14]. In the MG, glucose can be used to synthesize nonessential AA (NEAA). Restriction of glucose may limit the availability of NEAA in the bovine MG and may cause decreased milk protein synthesis [15]. In addition, the uptake of some NEAA into the bovine MG is substantially lower than their output in milk protein [16, 17]. The conversion of essential AA (EAA) to NEAA in the bovine MG is likely affected by the availability of glucose [18]. Moreover, the AMP-activated protein kinase (AMPK) plays an essential role in cellular energy sensing [19] and mTOR activation [20]. This signaling pathway may be involved in regulating the effect of local MG glucose supply (LMGS) on AA sensing and utilization in the lactating MG [21, 22]. However, our knowledge is limited on nutrient sensing via the AMPK and mTOR pathways in the MG.

Thus, the objective of this study was to determine the role of AMPK-mTOR pathway in AA sensing and utilization by directly infusing different dosages of glucose into the MG of dairy goats via the external pudendal artery (EPA) [23]. Our study involved direct and precise manipulation of the glucose influx into the MG relative to infusions from the abomasum or peripheral routes. Our study also provided a few reported parameters of milk amino acid profile in the dairy goats.

## Materials and methods

### Animals and experimental design

All experimental procedures were approved by the Animal Use and Care Committee of Zhejiang University (Hangzhou, China). The details of animals, catheterization, and treatments used in Exp.1 have been described previously [5]. Briefly, six lactating Guanzhong dairy goats (aged 3 years; days-in-milk: 113 ± 6; and BW: 43.6 ± 3.0 kg) were used. Milk yield of the animals was 1.47 ± 0.05 kg/d before the experiment began. Goats were fitted with catheters at the EPA and allowed to recover for 4 weeks [5] before being assigned to one of six dosages of glucose infusion (0, 20, 40, 60, 80, and 100 g/d] through the EPA catheter in a 6 × 6 Latin square design with repeated measures over 12-day periods consisting of 7 treatment days where glucose was infused each day for 5 h, followed by 5 transition days without glucose infusions. For each glucose treatment day, glucose was infused continuously from 12:00 to 17:00 for 5 h at a speed of 2.00 mL/min into the EPAs using syringe infusion pumps (Smiths WZS-50F6, Smiths Medical Instrument, Zhejiang, China). The goats were kept in individuals pens and milked 3 times a day (06:30, 10:00, and 19:00) using a portable milking machine. All goats were fed the same ration (Additional file 1: Table S1), providing 81% of the energy requirement and containing forage and pelleted concentrates at 07:00, 11:00, and 17:30. Feed was adjusted to allow for 5% orts. There were two reasons to provide animals with 81% of the energy requirements only: 1) the LMGS should have the largest effects on mammary AA sensing and utilization and milk protein synthesis under conditions of energy shortage, and 2) in Chinese dairy goat farms, energy shortage is generally considered common so the experiment was likely to be representative of the natural feeding conditions.

In the Exp.2, the same six catheterized, lactating dairy goats used in Exp.1 were infused via the EPA with three dosages of glucose [0 (low dose, LDG), 60 (optimal dose, MDG), and 100 (high dose, HDG) g/d glucose] in a 3 × 3 replicated Latin square design (replicate = 2). Identification of these dosages was based on the milk yield results from Exp.1, some of which were described elsewhere [5]. Briefly, LDG caused the lowest milk yield among all glucose infusion dosages; MDG resulted in the highest milk yield among all glucose infusion dosages; vice versa, HDG reduced milk yield when compared with MDG. The daily management of experimental goats, including glucose infusion, milking, and feeding, was similar to the procedures in Exp.1.

### Sampling and analyses

In Exp.1, feed samples were collected daily to analyze nutrient composition, as shown in Additional file 1: Table S1. Milk yield was recorded in the last 3 d of the treatment days, and milk samples were taken daily for immediate determination of milk protein or stored at − 20 °C for the subsequent analysis of 6 major milk proteins (α_S1_-casein, α_S2_-casein, β-casein, κ-casein, α-lactalbumin, and β-lactoglobulin) by reverse-phase HPLC (Agilent 1100; Agilent Technologies, Inc., Santa Clara, CA, USA) [24] and the milk protein AA profile after HCl hydrolysis. The major milk proteins in the milk samples including sample preparation, dilution and calibration curves were determined according to a published study [24]. At 06:30, 10:00, and 19:00, blood samples (5 mL) were collected from the EPA and mammary vein, and then immediately centrifuged at 3,000×*g* for 15 min at 4 °C to obtain the plasma. The plasma samples from 3 time points were pooled according to sample sites to analyze the AA profiles. Both the AA profiles in plasma and milk protein were measured by an Automatic AA Analyzer (Hitachi High-technologies Corporation, Tokyo, Japan).

Mammary tissue was sampled by biopsy in Exp.2 after the 19:00 milking on the last day of infusion. The mammary tissues were collected from the middle area of left udder in each period. The biopsy procedures were as follows: after milking, ketamine hydrochloride (4 mg/kg BW) and xylazine hydrochloride (0.5 mg/kg BW) were injected to anesthetize the goats. After cutting the skin of the MG, a sterile biopsy needle (14 G × 16 cm, Bard Peripheral Vascular, Inc., Tempe, AZ, USA) was inserted into the MG to approximately 9 cm to collect the mammary parenchyma. These samples were taken from the neighboring area (middle of the udder) to avoid previously biopsied areas. The tissue was immediately placed in sterile cryogenic vials and stored in liquid nitrogen.

### RNA extraction and real-time PCR analysis

Total RNA extraction and quality testing (OD_260_/OD_280_ ranged between 1.8 and 2.0 and RIN was greater than 7) followed the method described in a previous study [25]. The cDNA was synthesized to analyze the mRNA abundance of the selected genes following the method described previously [26]. The ubiquitously expressed transcript, mitochondrial ribosomal protein L39, and ribosomal protein S9 were used as reference genes after assessing their qualification using geNorm [27]. The sequences of primers for the analyzed genes [activating transcription factor 4 (*ATF4*), β-lactoglobulin (*BLG*), α_S2_-casein (*CSN1S2*), β-casein (*CSN2*), κ-casein (*CSN3*), eukaryotic translation elongation factor 1 α 1 (*EEF1A1*), eukaryotic translation elongation factor 2 (*EEF2*), eukaryotic translation initiation factor 4E binding protein 1 (*EIF4EBP1*), general control nonderepressible 2 (*GCN2*), α-lactalbumin (*LALBA*), *MTOR*, stress protein P8 (*NUPR1*), protein kinase AMP-activated catalytic subunit alpha 1 (*PRKAA1*), and ribosomal protein S6 kinase β-1 (*RPS6KB1*)] are listed in Additional file 1: Table S2. The relative change in the mRNA for each individual gene was calculated by the method according to Rao et al. [28].

### Signaling protein analysis

Total protein was extracted from tissues using RIPA lysis buffer with phenylmethylsulfonyl fluoride (1 mmol/L, Meibiao Biotechnology Co., Jiangsu, China). Approximately 20 mg of tissues were lysed into 150 μL of RIPA lysis buffer. After lysis, samples were centrifuged at a speed of 14,000×*g* for 5 min (4 °C). The supernatant was collected for determination of signaling proteins including mTOR, p-mTOR, AMPK, and p-AMPK using ELISA (goat mTOR ELISA kit, goat p-mTOR ELISA kit, goat AMPK ELISA kit, goat p-AMPK ELISA kit, Meibiao Biotechnology Co., Jiangsu, China). The primary antibodies were all mouse monoclonal antibodies and are specific for the respective goat proteins (Additional file 1: Table S3). The source of secondary antibodies was horseradish peroxidase coupled rabbit anti-mouse IgG H&L (Meibiao Biotechnology Co., Jiangsu, China; Additional file 1: Table S3). The standard curves are provided in the Additional file 1: Figure S1.

### Calculations

The mammary plasma flow (MPF) was estimated using the Fick principle, assuming the 100% transfer of free phenylalanine + tyrosine from plasma into milk protein [29], with an allowance of 3.5% contribution from blood-borne proteins [30]: MPF (L/d) = (milk Phe + Tyr) (g/d) × 0.965/[arterial − venous difference of (Phe + Tyr) (g/L)].

All individual AA supplies to the MG were calculated using AA concentrations from pooled samples from EPA by the following equation [14]: AA supply (mmol/d) = AA concentration in the EPA (mmol/L) × MPF (L/d). Mammary uptake of AA was calculated from measurements from pooled samples from EPA and mammary vein by the following equation [14]: mammary uptake of AA (mmol/d) = [plasma arterial − venous differences (mmol/L)] × MPF (L/d). Mammary clearance of AA was calculated by the following equation: Mammary clearance of AA (L/h) = [arterial − venous difference (mmol/L) × MPF (L/h)]/venous concentration (mmol/L). The uptake to output ratio (U:O) of AA was calculated by the following equation: U:O of AA = mammary uptake of AA (mmol/d)/[AA output in milk (mmol/d)].

### Statistical methods

Homogeneity of variances was examined by Levene statistics, and deviances from normality were examined by the Kolmogorov-Smirnoff test with Lilliefors correction. All data yielded *P* > 0.05 for the homogeneity of variances test and *P* > 0.05 for the normality test, indicating that the variances were homogenous and normally distributed and allowed for conducting parametric testing. All data were analyzed using the MIXED model (SAS Inst. Inc., Cary, NC) with a 6 × 6 Latin square design in Exp.1 and with a 3 × 3 replicated Latin square design (replicate = 2) in Exp.2. Treatment, goat, period, and residual effects were considered as the sources of the variation. The treatment and period were the fixed variables. The individual goat was considered as the random variable. The residual effect was used to test the significance of the treatment, goat, and period. Differences between the treatments were analyzed by orthogonal polynomial contrast with linear, quadratic, and cubic effects in Exp.1 and with linear and quadratic effects in Exp.2. The results are given as least square means with mean square errors. The effect was defined as significant at *P* ≤ 0.05 and as a tendency at 0.05 < *P* ≤ 0.10.

## Results

### AA profile and major protein components in milk

Composition and milk protein yield was not significantly changed (*P* > 0.10) by different glucose LMGS (Additional file 1: Table S4). No significant changes were observed in the individual AA or grouped AA, including EAA, NEAA, and branched-chain AA, in the milk of goats with different LMGS (Table 1, *P* > 0.10). Increasing the LMGS only showed a tendency of quadratic effects for a few individual AA, including His, Thr, Ala, Cys, and Gly, in milk. However, increasing the LMGS changed the total AA in milk in a quadratic manner (*P*-quadratic = 0.02), where they increased with infusions of 0 to 60 g/d and then decreased with infusions of 60 to 100 g/d. The effects of LMGS on milk protein compositions are presented in Table 2. Quadratic changes were observed in the percentage and yield of α_S2_-casein and α-lactalbumin (*P*-quadratic ≤ 0.05) in response to the increased LMGS, with the highest percentage and yield of α_S2_-casein and α-lactalbumin at the infused dose of 60 g/d. α_S1_-Casein was not detected in the milk of these dairy goats.

**Table 1:** Effects of increasing mammary gland glucose supply through external pudendal artery on milk protein AA profiles in lactating dairy goats

Item^1^	Infused glucose, g/d						SEM	*P*-value		
	0	20	40	60	80	100		Linear	Quadratic	Cubic
Essential AA, mmol/L										
Arg	6.36	6.78	7.81	6.94	5.73	5.62	1.15	0.44	0.19	0.31
His	5.33	6.32	6.92	6.22	5.81	5.54	0.73	0.66	0.08	0.28
Ile	9.91	10.2	11.7	9.9	10.3	10.1	0.95	0.98	0.35	0.23
Leu	23.2	23.9	24.3	25.3	22.4	21.7	2.88	0.20	0.47	0.26
Lys	15.6	15.8	16.2	16.3	15.9	15.7	1.59	0.85	0.31	0.27
Met	4.29	4.67	5.41	4.33	4.58	4.39	0.51	0.94	0.34	0.45
Phe	14.2	13.9	14.7	15.4	12.8	13.5	1.66	0.17	0.14	0.20
Thr	16.1	17.2	17.8	19.3	14.7	14.1	1.57	0.07	0.06	0.15
Val	17.6	16.8	17.8	18.1	17.1	15.6	1.75	0.70	0.45	0.26
Non-essential AA, mmol/L										
Ala	14.1	13.5	18.4	16.1	14.6	12.7	3.58	0.64	0.08	0.29
Asx	16.5	16.3	18.2	19.1	17.7	16.5	1.76	0.55	0.25	0.53
Cys	2.96	3.54	4.46	4.13	3.01	2.98	0.68	0.93	0.07	0.36
Glx	44.2	44.6	42.2	44.9	43.7	42.1	4.06	0.27	0.53	0.13
Gly	6.88	8.96	13.8	13.6	8.84	8.12	2.31	0.79	0.08	0.23
Pro	20.2	20.7	23.2	23.5	20.4	19.5	2.77	0.93	0.37	0.25
Ser	18.8	22.2	21.1	24.3	19.2	18.5	4.21	0.82	0.31	0.70
Tyr	10.6	11.4	11.6	12.2	11.8	12.4	1.12	0.97	0.55	0.28
Essential AA, mmol/L	113	116	123	122	109	106	6.51	0.29	0.13	0.63
Non-essential AA, mmol/L	134	141	153	158	139	133	10.5	0.39	0.13	0.68
Group-1 AA, mmol/L	34.4	36.3	38.6	38.2	35	35.8	4.29	0.59	0.36	0.28
Group-2 AA, mmol/L	88.8	90.7	95.6	95.8	86.1	82.8	5.72	0.44	0.25	0.42
Branched-chain AA, mmol/L	50.7	50.9	53.8	53.3	49.8	47.4	3.98	0.28	0.46	0.37
Total AA, mmol/L	247	257	276	280	249	239	11.4	0.60	0.02	0.43

^1^ Group-1 AA: His, Met, Phe, and Tyr; Group-2 AA: Arg, Ile, Leu, Lys, Thr, and Val; Branched-chain AA: Ile, Leu, and Val. Glx: glutamate + glutamine. Asx: aspartate + asparagine

**Table 2:** Effects of increasing mammary glucose supply on milk protein composition in lactating dairy goats

Items	Infused glucose, g/d						SEM	*P*-value		
	0	20	40	60	80	100		Linear	Quadratic	Cubic
Protein profile, % of milk protein										
α_S2_-casein	10.6	11.3	12.8	14.3	12.4	11.7	1.05	0.21	0.03	0.52
β-casein	55.5	52.5	55.0	52.7	54.4	54.2	1.86	0.85	0.53	0.66
κ-casein	16.6	17.5	15.5	15.0	14.9	15.9	1.00	0.11	0.29	0.15
α-lactalbumin	3.78	4.07	4.27	5.55	4.15	3.57	0.44	0.90	0.02	0.27
β-lactoglobulin	13.5	14.7	12.5	12.5	14.1	14.6	1.06	0.65	0.24	0.53
Protein fraction yield, g/d										
α_S2_-casein	2.81	3.17	3.88	4.66	3.44	3.58	0.28	0.03	< 0.01	0.77
β-casein	15.1	14.9	16.8	17.4	15.3	16.6	1.09	0.30	0.40	0.87
κ-casein	4.44	4.95	4.70	4.99	4.17	4.97	0.43	0.87	0.82	0.21
α-lactalbumin	1.01	1.19	1.30	1.84	1.17	1.12	0.15	0.43	< 0.01	0.48
β-lactoglobulin	3.60	4.19	3.76	4.07	3.96	4.45	0.33	0.18	0.80	0.31

### Supply of AAs to the MG

The AA concentrations in the EPA are provided in Additional file 1: Table S5. Quadratic changes were observed in Lys (*P*-quadratic < 0.01), Val (*P*-quadratic = 0.01), Met (*P*-quadratic = 0.01), and Glx (sum of glutamic acid and glutamin) (*P*-quadratic = 0.04) with increasing LMGS. The mammary artery supply of AA in response to LMGS are presented in Additional file 1: Table S6. Most of EAA, including Arg, Lys, Met, Thr, and Val, changed quadratically (*P*-quadratic ≤0.05), increasing from 0 to 60 g/d of LMGS and then decreasing from 60 to 100 g/d of LMGS. The NEAA, Glx, Ser, and Tyr changed in a quadratic manner (*P*-quadratic ≤ 0.05), with maximum levels at the infusion of 60 g/d. Increasing the LMGS quadratically changed the mammary artery supply of EAA, NEAA, group-1 AA, group-2 AA, branched-chain AA, and total AA (*P*-quadratic ≤ 0.05), showing increased effects from 0 to 60 g/d of LMGS and then decreased effects from 60 to 100 g/d of LMGS.

### AA uptake and utilization in the MG

As shown in Table 3, mammary uptake of EAA, NEAA, group-2 AA, branched-chain AA, and total AA were quadratically changed (*P*-quadratic ≤ 0.05) with the increased LMGS, increasing at doses from 0 to 60 g/d and decreasing at doses from 60 to 100 g/d. Quadratic changes of EAA, including Arg, Ile, Leu, Lys, Met, Phe, Thr, and Val (*P*-quadratic ≤ 0.05), were observed. Mammary uptake of most NEAA did not change with the LMGS (*P* > 0.10), except for Cys (*P*-quadratic < 0.01), Glx (*P*-quadratic < 0.01) and Ser (*P*-quadratic = 0.04), which showed quadratic changes.

**Table 3:** Effects of increasing mammary glucose supply on the mammary gland uptake of AA in lactating dairy goats^1^

Items^2^	Infused glucose, g/d						SEM	*P*-value		
	0	20	40	60	80	100		Linear	Quadratic	Cubic
Essential AA, mmol/d										
Arg	12.3	14.5	15.9	23.6	14.3	9.32	5.01	0.96	0.06	0.78
His	10.2	8.18	12.7	17.2	17.4	7.20	5.24	0.81	0.22	0.15
Ile	15.7	19.2	25.2	32.4	17.9	14.3	5.32	0.95	0.02	0.68
Leu	16.7	21.4	27.2	31.8	21.1	19.5	4.13	0.32	< 0.01	0.74
Lys	11.8	13.5	22.9	26.9	17.7	14.2	1.86	0.39	< 0.01	0.50
Met	2.97	3.96	6.38	7.52	5.21	3.34	1.18	0.80	< 0.01	0.92
Phe	10.3	11.9	13.9	16.7	10.1	11.2	1.98	0.76	0.04	0.81
Thr	11.2	15.4	16.7	18.8	15.1	13.4	2.29	0.57	0.02	0.28
Val	16.1	16.7	20.5	24.6	20.8	15.9	2.21	0.39	< 0.01	0.24
Non-essential AA, mmol/d										
Ala	14.6	13.2	20.3	15.5	11.7	10.3	3.34	0.51	0.58	0.91
Asx	2.88	1.42	2.05	2.04	4.41	1.52	0.87	0.99	0.74	0.13
Cys	3.06	4.38	7.14	8.03	4.58	3.53	1.22	0.36	< 0.01	0.64
Glx	19.2	25.3	38.8	44.9	32.3	20.2	4.63	0.26	< 0.01	0.90
Gly	4.55	5.04	11.1	9.95	8.01	5.83	2.89	0.67	0.08	0.87
Pro	9.20	11.5	13.6	11.9	14.2	9.3	4.55	0.69	0.25	0.25
Ser	11.5	17.1	22.3	23.5	13.2	12.8	4.84	0.81	0.03	0.57
Tyr	8.80	10.6	10.9	12.1	9.50	10.1	1.82	0.69	0.15	0.67
Essential AA, mmol/d	107	125	161	200	140	108	23.2	0.49	< 0.01	0.26
Non-essential AA, mmol/d	73.8	88.5	126	128	97.9	73.6	14.2	0.58	0.04	0.62
Group-1 AA, mmol/d	32.3	34.6	43.9	53.5	42.2	31.8	8.25	0.70	0.07	0.86
Group-2 AA, mmol/d	83.8	100	128	158	107	86.6	19.9	0.65	0.01	0.50
Branched-chain AA, mmol/d	48.5	57.3	72.9	88.8	59.8	49.7	10.1	0.70	< 0.01	0.59
Total AA, mmol/d	181	213	288	327	238	182	33.4	0.59	< 0.01	0.45

^1^ Mammary gland uptake of AA (mmol/d) = (arterial − venous difference) (mmol/L) × mammary gland plasma flow (L/d)

^2^ Glx: glutamate + glutamine; Asx: aspartate + asparagines; Group-1 AA: His, Met, Phe, and Tyr; Group-2 AA: Arg, Ile, Leu, Lys, Thr, and Val; Branched-chain AA: Ile, Leu, and Val

Increasing LMGS from 0 to 60 g/d resulted in the quadratically increased clearance rate of Cys (*P*-quadratic < 0.01), Glx (*P*-quadratic ≤ 0.05), and Lys (*P*-quadratic < 0.01), but decreased clearance rate of Cys, Glu, and Lys when LMGS reached at 100 g/d (Additional file 1: Table S7).

The effect of the LMGS on the U:O of AA is shown in Table 4. Quadratic changes (*P*-quadratic ≤ 0.05) in Cys, Glx, Leu, Lys, and Met were observed, with increased changes at doses from 0 to 60 g/d of LMGS and decreased changes at doses from 60 to 100 g/d of LMGS. The U:O ratios of EAA (*P*-quadratic = 0.02) and Group-2 AA (*P*-quadratic ≤ 0.01) were also quadratically changed, following the same trends.

**Table 4:** Effects of increasing mammary glucose supply on the AA uptake to output ratio in the mammary gland of lactating dairy goats^1^

Item^2^	Infused glucose, g/d						SEM	*P*-value		
	0	20	40	60	80	100		Linear	Quadratic	Cubic
Essential AA										
Arg	2.79	2.85	2.50	3.86	3.22	2.19	1.32	0.87	0.52	0.59
His	2.77	1.73	2.26	3.14	3.86	1.71	1.03	0.92	0.55	0.12
Ile	2.29	2.51	2.65	3.72	2.24	1.87	0.74	0.55	0.22	0.47
Leu	1.04	1.19	1.38	1.43	1.21	1.19	0.12	0.37	0.04	0.69
Lys	1.09	1.14	1.74	1.88	1.43	1.19	0.16	0.33	< 0.01	0.16
Met	1.00	1.13	1.45	1.97	1.47	1.00	0.31	0.91	0.04	0.70
Phe	1.05	1.14	1.16	1.23	1.02	1.09	0.31	0.95	0.68	0.77
Thr	1.01	1.19	1.15	1.11	1.32	1.25	0.14	0.88	0.29	0.52
Val	1.32	1.33	1.42	1.54	1.57	1.34	0.11	0.95	0.30	0.44
Non-essential AA										
Ala	1.50	1.30	1.36	1.09	1.03	1.07	0.35	0.13	0.73	0.85
Asx	0.25	0.12	0.14	0.12	0.32	0.12	0.11	0.86	0.96	0.34
Cys	1.49	1.65	1.97	2.21	1.96	1.56	0.15	0.67	< 0.01	0.21
Glx	0.63	0.76	1.13	1.14	0.95	0.63	0.12	0.43	< 0.01	0.87
Gly	0.96	0.75	0.99	0.83	1.17	0.95	0.25	0.53	0.34	0.84
Pro	0.66	0.74	0.72	0.58	0.90	0.63	0.32	0.69	0.86	0.55
Ser	0.88	1.03	1.30	1.10	0.89	0.91	0.23	0.73	0.29	0.71
Tyr	1.20	1.24	1.16	1.13	1.04	1.07	0.28	0.89	0.34	0.85
Essential AA	1.38	1.44	1.62	1.86	1.65	1.35	0.14	0.82	0.02	0.57
Non-essential AA	1.74	1.81	1.13	1.46	1.38	1.82	0.39	0.78	0.40	0.62
Group-1 AA	1.35	1.27	1.40	1.59	1.55	1.17	0.47	0.73	0.73	0.41
Group-2 AA	1.36	1.48	1.65	1.87	1.60	1.38	0.13	0.59	< 0.01	0.65
Branched-chain AA	1.38	1.50	1.67	1.89	1.55	1.38	0.38	0.87	0.10	0.78
Total AA	1.06	1.11	1.28	1.33	1.23	1.00	0.31	0.90	0.22	0.74

^1^ AA uptake to output ratio = Mammary gland uptake of AA (mmol/d)/[AA output in milk (mmol/d)]

^2^ Glx: glutamate + glutamine; Asx: aspartate + asparagines; Group-1 AA: His, Met, Phe, and Tyr; Group-2 AA: Arg, Ile, Leu, Lys, Thr, and Val; Branched-chain AA: Ile, Leu, and Val

### Expression of genes involved in the milk protein synthesis and signaling pathways in the MG

**Fig. 1:**
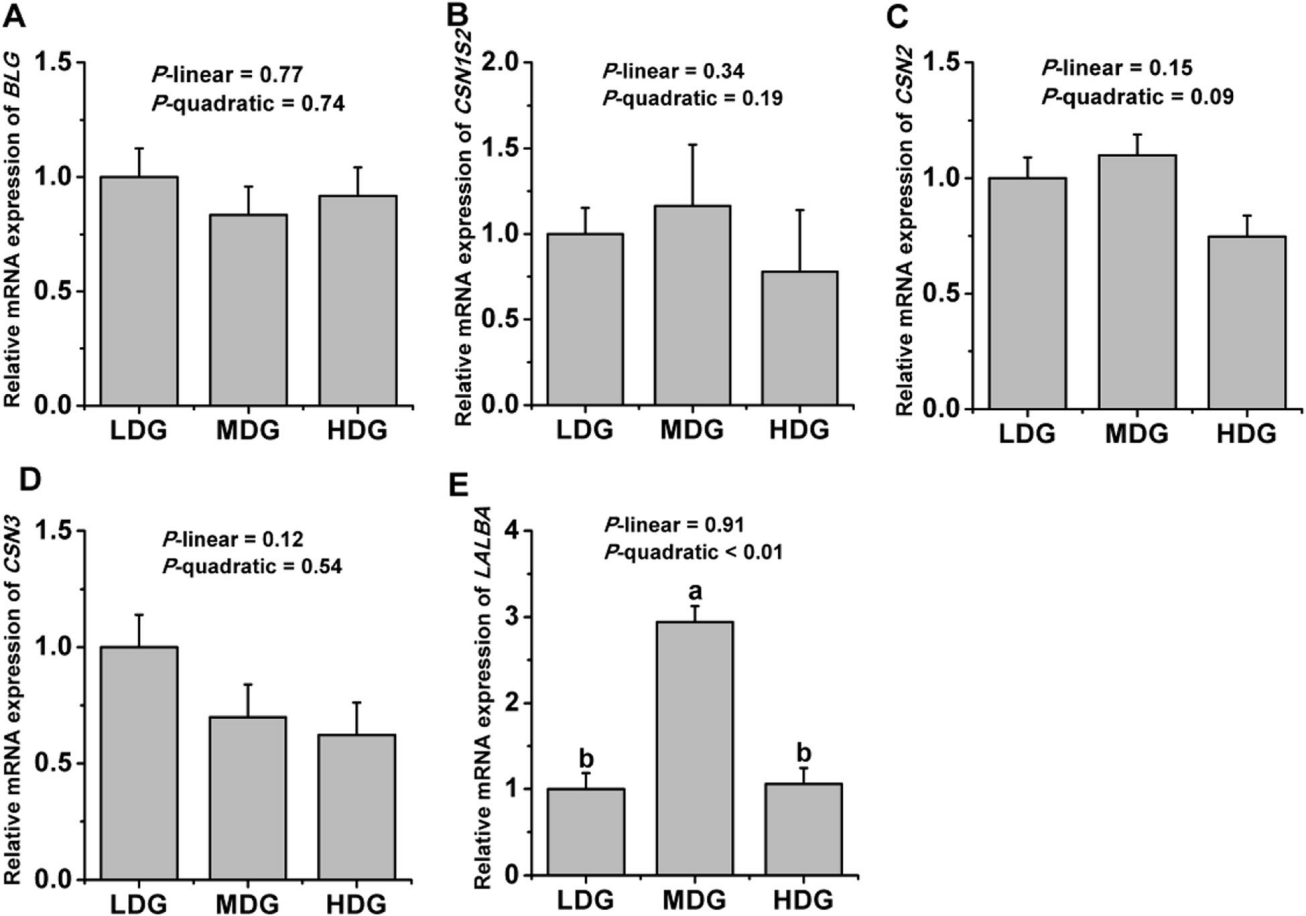
Relative mRNA expression of genes encoding the main milk proteins in the mammary gland of lactating goats infused with low (LDG, 0 g/d), middle (MDG, 60 g/d) or high (HDG, 100 g/d) glucose dosages through the external pudendal artery in Exp.2. Error bars represent pooled SEM. ^a,b,c^Values without a common letter for a gene differ (*P* ≤ 0.05). A *BLG* = β-lactoglobulin; B *CSN1S2* = α_S2_-casein; C *CSN2* = β-casein; D *CSN3* = κ-casein; E *LALBA* = α-lactalbumin

**Fig. 2:**
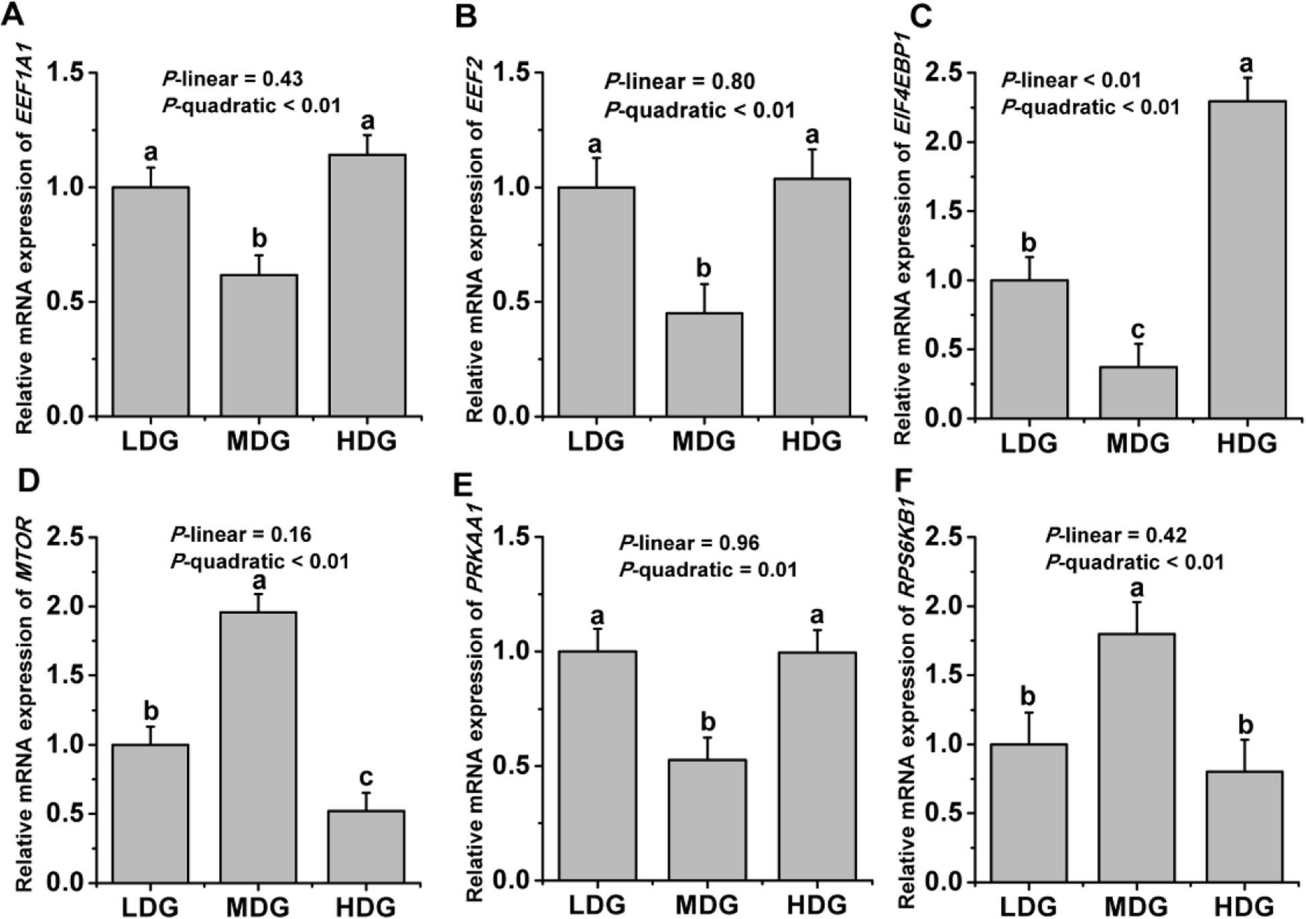
Relative mRNA expression of genes involved in the AMPK-mTOR pathway in lactating mammary glands of dairy goats locally treated with low (LDG, 0 g/d), middle (MDG, 60 g/d) or high (HDG, 100 g/d) glucose dosages in Exp.2. Error bars represent the pooled SEM. ^a,b,c^Values without a common letter for a gene differ (*P* ≤ 0.05). A *EEF1A1* = eukaryotic translation elongation factor 1 α 1; B *EEF2* = eukaryotic translation elongation factor 2; C *EIF4EBP1* = eukaryotic translation initiation factor 4E binding protein 1; D *MTOR* = mechanistic target of rapamycin; E *PRKAA1* = protein kinase AMP-activated catalytic subunit alpha 1; F *RPS6KB1* = ribosomal protein S6 kinase β-1

As shown in Fig.1, mRNA expression of *LALBA* (*P*-quadratic < 0.01) showed a quadratic increase with increasing LMGS and had the greatest expression at the glucose infusion of 60 g/d, whereas no differences were detected in the mRNA levels of *CSN1S2* (*P* > 0.05), *CSN3* (*P* > 0.05), and *BLG* (*P* > 0.05) between all treatments. A tendency towards quadratic response of *CSN2* mRNA expression (*P*-quadratic = 0.09) to increasing LMGS was found with a highest *CSN2* mRNA expression at a glucose infusion rate of 60 g/d. The mRNA expression of genes involved in the AMPK-mTOR pathway is shown in Fig.2. Quadratic decreases of mRNA expression of *PRKAA1* (*P*-quadratic = 0.01), *EIF4EBP1* (*P*-quadratic < 0.01), *EEF1A1* (*P*-quadratic < 0.01), and *EEF2* (*P*-quadratic < 0.02) were found with increasing infused LMGS, with lowest mRNA expression at the infusion level of 60 g/d. However, quadratic increases of higher mRNA expression of *MTOR* (*P*-quadratic < 0.01) and *RPS6KB1* (*P*-quadratic < 0.01) were found with increasing infused LMGS, with highest mRNA expression at the infusion rate of 60 g/d.

**Fig. 3:**
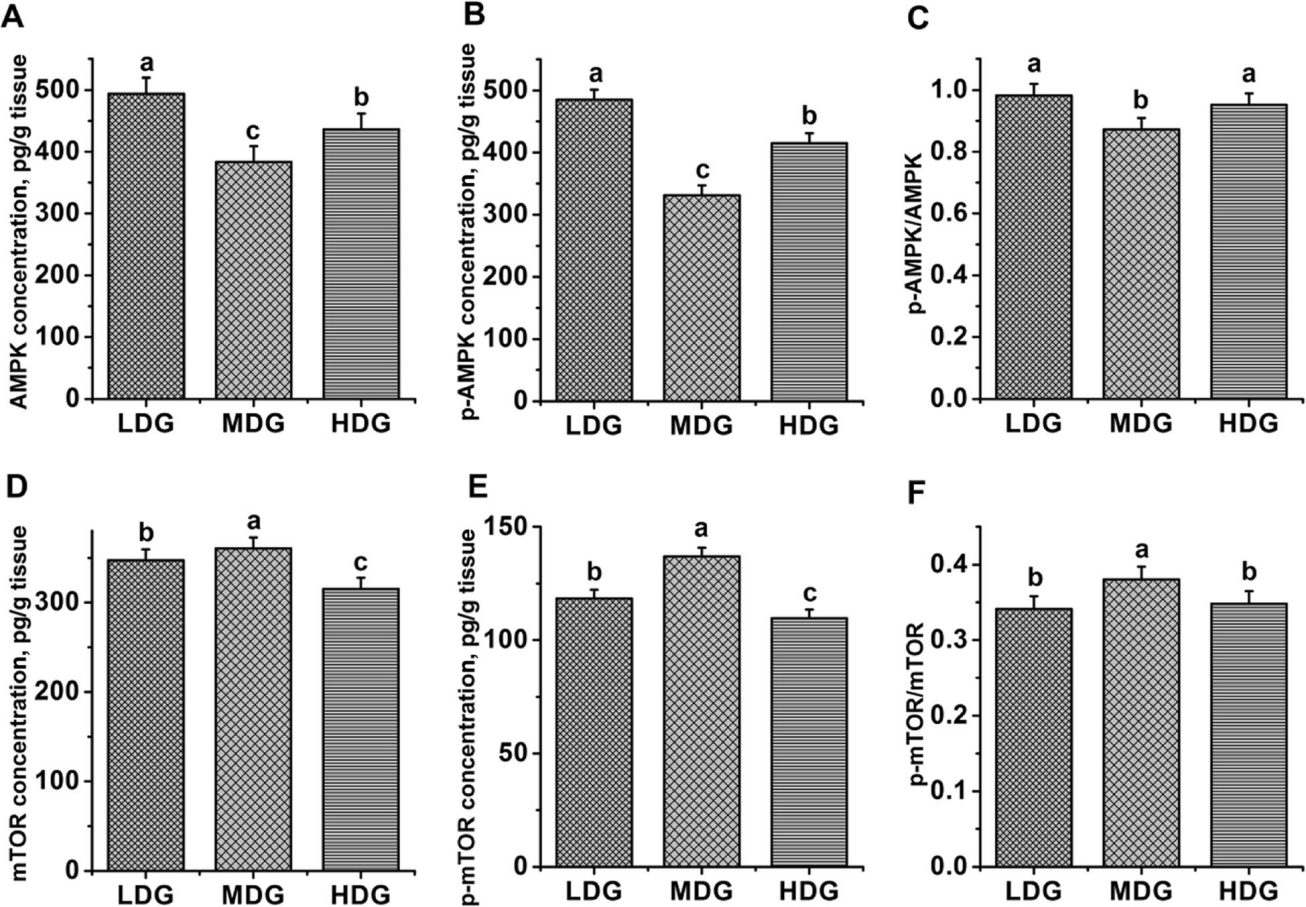
Protein abundance and phosphorylation state of AMPK and mTOR in lactating mammary glands of dairy goats locally treated with low (LDG, 0 g/d), middle (MDG, 60 g/d) or high (HDG, 100 g/d) glucose dosages in Exp.2. Error bars represent the pooled SEM. ^a,b,c^Values without a common letter differ (*P* ≤ 0.05). A AMPK = AMP-activated protein kinase; B p-AMPK = phosphorylated AMP-activated protein kinase; C p-AMPK/AMPK = the ratio of phosphorylated AMP-activated protein kinase to AMP-activated protein kinase; D mTOR = mechanistic target of rapamycin; E p-mTOR *=* phosphorylated mechanistic target of rapamycin; F p-mTOR/mTOR = the ratio of phosphorylated mechanistic target of rapamycin to phosphorylated mechanistic target of rapamycin

The protein abundance and phosphorylation state of AMPK and mTOR signaling proteins in the MG are shown in Fig.3. Quadratic increases in total mTOR (*P*-quadratic < 0.01) and p-mTOR levels (*P*-quadratic < 0.01) as well as the ratio of p-mTOR to mTOR (*P*-quadratic < 0.01) were observed when increasing LMGS was infused, with highest levels at the glucose infusion rate of 60 g/d. Quadratic decreases of total AMPK (*P*-quadratic < 0.01) and p-AMPK levels (*P*-quadratic < 0.01) as well as the ratio of p-AMPK to AMPK (*P*-quadratic < 0.01) were observed when increasing LMGS was infused, with the lowest ratio at the glucose infusion of 60 g/d.

**Fig. 4:**
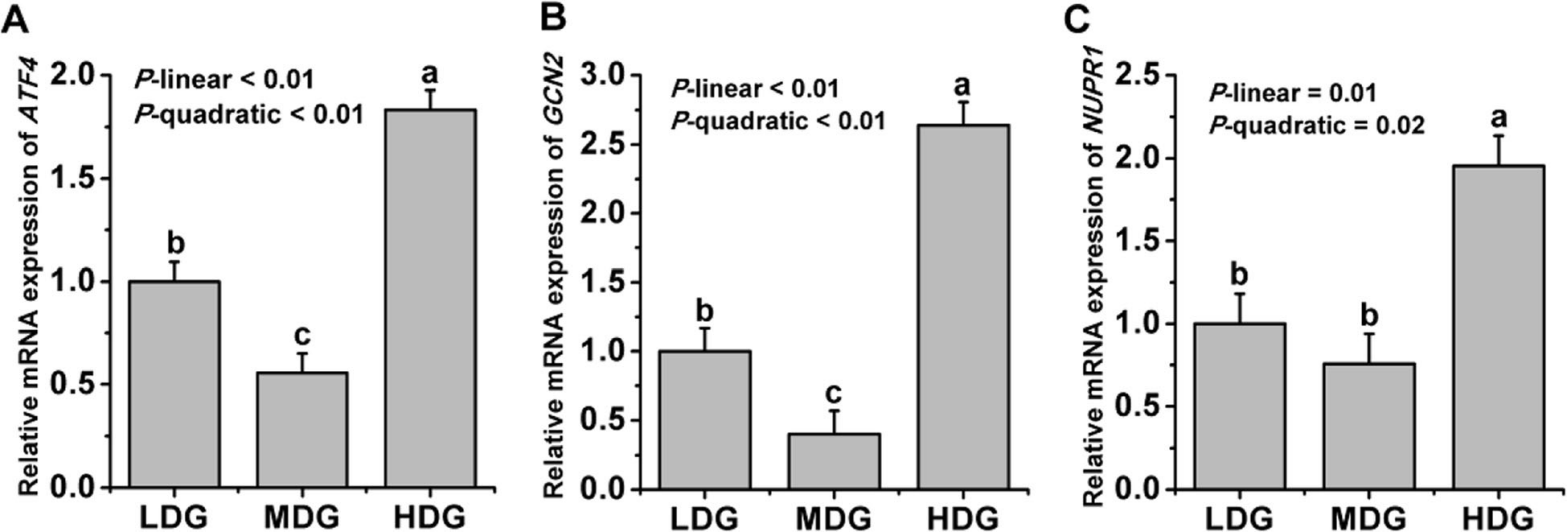
Relative mRNA expression of genes involved in amino acid response pathways in lactating mammary glands of dairy goats locally treated with low (LDG, 0 g/d), middle (MDG, 60 g/d) or high (HDG, 100 g/d) glucose dosages in Exp.2. Error bars represent pooled SEM. ^a,b,c^Values without a common letter were significantly different (*P* ≤ 0.05). A *ATF4* = activating transcription factor 4; B *GCN2* = general control nonderepressible 2; C *NUPR1* = stress protein P8

The expression of two genes involved in the AA sensory pathways is shown in Fig.4. Quadratic decrease of *GCN2* (*P*-quadratic < 0.01), *ATF4* (*P*-quadratic < 0.01) and *NUPR1* (*P*-quadratic = 0.02) were found when increasing LMGS was infused, with lowest mRNA expression at the glucose infusion rate of 60 g/d.

## Discussion

### Effects of LMGS on mammary AA utilization

Mammary AA availability is considered as the main limiting factor for milk protein synthesis [31, 32]. In this study, the supply of most EAA and glucogenic NEAA (Glx and Ser) to the MG was improved when the LMGS was increased from 0 to 60 g/d. These changes likely resulted from the reduced glucogenesis rates for these AA in the liver with an unchanged dry matter intake which was reported in previous work [5]. Rulquin et al. also showed that the intestinal glucose supply affects circulating AA to the MG [33]. The milk urea concentration linearly decreased with increasing LMGS in our previous study, indicating that when more glucose is available, less AA are catabolized and a greater proportion of AA is used to synthesize protein in the MG [5]. However, the U:O of the EAA, such as Lys and Leu, still increased in response to increasing LMGS from 0 to 60 g/d, indicating that increased synthesis of NEAA or other metabolites from these EAA are still required in the MG to support the increased milk yield in goats infused with 0–60 g/d glucose [5]. Moreover, milk yield and milk protein yield tended to decrease when the LMGS was increased from 60 to 100 g/d [5]. This decrease was consistent with the decreased supply of AA (particularly EAA) and mammary uptake in these animals. Thus, it appears that when a high dosage of glucose was given, AA availability to the MG was inhibited, and may have mainly resulted from a trend of decreased MPF in these animals observed in both our previous study [5] and another study [34]. In addition, our previous study showed that high dosage of glucose resulted in acute glycolysis in mammary epithelial cells (MEC), accumulated reactive oxygen species and decreased the antioxidant capacity of MEC [5]. Thus, an optimal supply of glucose can lead to greater mammary artery supply and utilization of AA compared with those at lower or even higher glucose supply. Moreover, the current work also contributes to the understanding of a few reported milk AA profiles in the dairy goats.

### Effects of LMGS on mammary AA sensing

The AA availability is sensed by cells through nutrient regulatory signaling pathways [6]. Previous studies have demonstrated that mTOR plays an important role in linking AA abundance to several bioprocesses, including cellular stress and energy status [6, 35]. In addition, GCN2, a serine/threonine protein kinase, converges on PHA-4/FoxA as well as related downstream pathway to keep the cells alive under AA deficiency condition [36]. The responses of GCN2 to AA deficiency are initiated by binding to uncharged tRNAs and activated EIF2 and ATF4, which function together as major response to restore AA metabolism and homeostasis [22, 37]. In our studies, the mRNA expression and total protein abundance of mTOR as well as the p-mTOR concentration and the ratio of p-mTOR to mTOR were all increased in the MG of the MDG group compared with the LDG and HDG groups, coinciding with mammary AA supply, uptake and milk yield [5]. Lower mTOR pathway activation was observed in LDG and HDG animals, compared to animals in MDG group. In addition, reduced expression of *GCN2* in MDG animals compared with LDG and HDG goats may suggest an important regulatory role of *GCN2* in glucose-mediated intracellular AA fluctuations. Consistently, the lower mRNA expression of *ATF4* in MDG goats relative to LDG and HDG goats and the higher mRNA expression of *ATF4* in HDG animals relative to LDG and MDG goats further support the role of the GCN2-EIF2-ATF4 pathway in sensing AA availability in this study. Furthermore, P8, the stress inducible protein coded by *NUPR1*, was highly expressed when the highest level of glucose was infused into the MG in the HGD group. Nutrient-mediated stress was likely induced by the high dosage of local glucose through the expression of *ATF4*-dependent *NUPR1*, which may in turn have inhibited protein synthesis by endoplasmic reticulum stress or redox stress [38, 39]. In summary, our results suggest that the mTOR pathway may be activated and GCN2-EIF2-ATF4 pathway inhibited in MECs when there is increased glucose and AA availability in the MG. On the other hand, high dosages of local glucose disrupt the AA availability in the MG, which can be sensed by the activation of the GCN2-EIF2-ATF4 pathway and stress proteins, and inhibition of the mTOR pathway.

### Response of α-lactalbumin to LMGS

AMPK is a cellular energy sensor [40, 41]. Zhang et al. showed that glucose deficiency activates the AMPK signaling pathway [42]. In this study, we observed that both LDG and HDG animals had higher mRNA abundance of *PRKAA1* (encoding the catalytic subunit of the 5′-prime-AMPK), AMPK abundance and p-AMPK levels in the MG than those of MDG animals. The higher AMPK expression could lead to reduced mRNA abundance of α-lactalbumin in MECs and milk α-lactalbumin content. The possible higher sensitivity of *LALBA* gene expression to LMGS compared to other milk proteins probably occurred because it is a part of lactose synthase, which catalyzes lactose synthesis from glucose [22]. The ratio of AMP to ATP within MECs could be influenced by the LMGS, with higher values in the LDG due to lower glucose availability and in the HDG due to increased glycolysis [5, 43, 44]. AMPK signaling became activated when the ratio of AMP/ATP was high. The expression of *LALBA* may be under the direct inhibition of AMPK, which can link lactose synthesis to optimal intracellular glucose availability [45–47]. Consistently, the yield of lactose [5] and α-lactalbumin in milk was higher in the MDG group compared to the LDG and HDG groups. We propose that the AMPK signaling changes may be at least partially responsible for the mTOR signaling changes as visible from the current study and data from another study [42].

### Response of α_S2_-casein to LMGS

Protein synthesis is largely affected by the availability and balance of AA [42]. For example, protein profiles synthesized under excess or deficiency of any AA in animal rations can be different from those of animals at adequate levels of dietary energy [48, 49]. Conde-Aguilera et al. showed that the supply of sulfur AA resulted in the high Cys content in the proteins of muscle, jejunum and ileum [50]. In our study, the supply and utilization of AA in the MG were affected by the LMGS, including the quadratic changes in mammary artery supply of Val, Met, Lys, Thr, Arg, Ser, Glx, and Tyr, the quadratic changes in mammary uptake of Cys, Glx, Ser and most EAA, the quadratic changes in U:O ratios of Met, Leu, Lys, Glx, and Cys, and the quadratic changes in mammary clearance rates of Lys, Glu, and Cys, with more pronounced changes at dosages from 0 to 60 g/d and decreased effects at dosages between 60 to 100 g/d. Taking all these changes into consideration, Lys and Glx consistently responded to the LMGS changes. Accordingly, the milk content and yield of α_S2_-casein also showed the same quadratic changes with increased changes at doses from 0 to 60 g/d of LMGS and decreased changes at doses from 60 to 100 g/d. The quadratic increase of milk content and yield of α_S2_-casein could be associated with quadratic increase in supplies of Lys and Glx at doses from 0 to 60 g/d of LMGS because the content of these two AA in α_S2_-casein (21.9%) was higher compared with that in β-casein (14.4%), κ-casein (10.4%), α-lactalbumin (13.4%), and β-lactoglobulin (17.8%).

## Conclusions

Compared to MDG goats, LDG and HDG goats had lower gene expression of *LALBA*, lower protein levels and activation of mTOR, lower mammary artery supply and utilization of AA, but higher protein levels and activation of AMPK. These changes may contribute to the lower concentrations of total milk AA, α_S2_-casein, α-lactalbumin as well as the yields of α_S2_-casein and α-lactalbumin in these goats. Moreover, excessive glucose may cause nutrient stress and induced the *GCN2* expression, which resulted in reduced total AA output into milk in HDG goats. Our findings provide new insights into nutrient interactions coordinated by the AMPK-mTOR pathway in the MG of dairy goats as well as other dairy ruminants.

## Supplementary information

**Additional file 1: Table S1.** Ingredient and chemical compositions of the basal diet fed to lactating dairy goats. **Table S2.** Primers used for quantitative real-time PCR. **Table S3.** Antibodies used in the signaling protein analysis. **Table S4.** Effects of increasing mammary glucose supply by external pudendal artery on composition and milk protein yield. **Table S5.** Effects of increasing mammary gland glucose supply through the external pudendal artery on AA concentration in external pudendal artery in lactating dairy goats. **Table S6.** Effects of increasing the mammary glucose supply on the AA supply to the mammary gland of lactating dairy goats. **Table S7.** Effects of increasing mammary gland glucose supply through external pudic artery on mammary clearance rate of amino acids (AA). **Figure S1.** Standard curve for the determination of signaling proteins.

## Data Availability

All data measured or analyzed during this work are available from the corresponding author upon reasonable request.

## Abbreviations

AA: Amino acid
AMPK: AMP-activated protein kinase;
ATF4: Activating transcription factor 4
BLG: β-lactoglobulin
CSN1S2: α_S2_-casein
CSN2: β-casein
CSN3: κ-casein
EAA: Essential amino acid
EEF1A1: Eukaryotic translation elongation factor 1 α 1
EEF2: Eukaryotic translation elongation factor 2
EIF4EBP1: Eukaryotic translation initiation factor 4E binding protein 1
EPA: External pudendal artery
GCN2: General control nonderepressible 2
HDG: High dose of glucose
LALBA: α-Lactalbumin
LDG: Low dose of glucose
LMGS: Local mammary glucose supply
MDG: Middle dose of glucose
MEC: Mammary epithelial cell
MPF: Mammary plasma flow
mTOR: Mechanistic target of rapamycin
NEAA: Nonessential amino acid
NUPR1: Stress protein P8
PRKAA1: Protein kinase AMP-activated catalytic subunit alpha 1
RPS6KB1: Ribosomal protein S6 kinase β-1
