# Supplementary material for: AMPK-mTOR pathway is involved in glucose-modulated amino acid sensing and utilization in the mammary glands of lactating goats
Source: J Anim Sci Biotechnol. 2020 Feb 14;11:32. doi: 10.1186/s40104-020-0434-6 (PMC7060552; doi:10.1186/s40104-020-0434-6)
Supplement: Supplementary file 1 — Additional file 1: Table S1. Ingredient and chemical compositions of the basal diet fed to lactating dairy goats. Table S2. Primers used for quantitative real-time PCR. Table S3. Antibodies used in the signaling protein analysis. Table S4. Effects of increasing mammary glucose supply by external pudendal artery on composition and milk protein yield. Table S5. Effects of increasing mammary gland glucose supply through the external pudendal artery on AA concentration in external pudendal artery in lactating dairy goats. Table S6. Effects of increasing the mammary glucose supply on the AA supply to the mammary gland of lactating dairy goats. Table S7. Effects of increasing mammary gland glucose supply through external pudic artery on mammary clearance rate of amino acids (AA). Figure S1. Standard curve for the determination of signaling proteins. [file 40104_2020_434_MOESM1_ESM.doc]

Supplementary Table S1. Ingredient and chemical compositions of the basal diet fed to lactating dairy goats.

| Item | Amount |
| --- | --- |
| Ingredient, % (DM basis) |  |
| Alfalfa hay | 20.0 |
| Peanut cane | 16.0 |
| Chinese wildrye grass hay | 32.0 |
| Corn grain | 15.4 |
| Wheat bran | 6.4 |
| Soybean meal | 3.8 |
| Canola meal | 2.6 |
| Salt | 1.6 |
| CaHPO_4_ | 0.6 |
| Premix*^1^* | 1.6 |
| Composition, % of DM |  |
| Dry matter | 88.4 |
| Organic matter | 90.9 |
| Crude protein | 11.3 |
| Ether extracts | 3.1 |
| Neutral detergent fiber | 34.3 |
| Acid detergent fiber | 24.1 |
| Non fibrous carbohydrates | 42.8 |
| NE_L_, MCal/kg | 1.1 |

*^1^* Formulated to provide (per kilogram of DM): for mineral: 65 g Mg, 3.2 g Cu, 6.5 g Fe, 27.8 g Mn, 25.6 g Zn, 35 mg Se, 78 mg Co, 0.3 g I, 55 g S; fat-soluble vitamins: 2000 k IU vitamin A, 350 k IU vitamin D_3_, 2.5 g vitamin E; water-soluble vitamins: 10 g niacin, and 20 g choline.

Supplementary Table S2. Primers used for quantitative real-time PCR.

| Gene | Gene Accession | Primers | Primer sequence (5' to 3') | Amplicon size (bp) | References |
| --- | --- | --- | --- | --- | --- |
| *ATF4* | XM_018048794.1 | F.455 | GTTCTCCTGCGACAAGGCTA | 213 | This study |
|  |  | R.667 | TCATCCAACGTGGCCAAGAG |  |  |
| *BLG* | EU573197.1 | F.60 | CGCTGTCTCAGCCCTCCACTC | 128 | This study |
|  |  | R.187 | GTCTGGGTGACGATGATGGCC |  |  |
| *CSN1S2* | NM_001285585.1 | F.168 | GGCCATTCATCCCAGAAAGGA | 122 | This study |
|  |  | R.289 | CTGGGGCAACTTCAGCAGAT |  |  |
| *CSN2* | XM_005681721.2 | F.189 | TCGGTGAGACTGTGGAAAGC | 136 | This study |
|  |  | R.324 | TGGGCAAAGGGGTGGATTTT |  |  |
| *CSN3* | NM_001285587.1 | F.83 | TATCCTGGCATTAACCCTGCC | 238 | This study |
|  |  | R.320 | CCTAACTGCAACTGGCTTTGC |  |  |
| *EEF1A1* | EF564269.1 | F.63 | TCCTGGCAAGCCTATGTGTG | 103 | This study |
|  |  | R.165 | TTTGATGACACCCACAGCGA |  |  |
| *EEF2* | XM_005682572.3 | F.1655 | CAAGCTGGTGGAAGGTCTGA | 186 | This study |
|  |  | R.1840 | CGGTACGAGACAACTGGGTC |  |  |
| *EIF4EBP1* | NM_001285589.1 | F.245 | CTAGCCCTACAGGCGATGAG | 108 | This study |
|  |  | R.352 | TGTCCATCTCAAACTGTGACTCT |  |  |
| *GCN2* | XM_018054469.1 | F.3773 | AGCTGACGAGGAGAGAAGTG | 240 | This study |
|  |  | R.4012 | ACTGGGGAATCTGTAACTTTATGC |  |  |
| *LALBA* | NM_001285635.1 | F.257 | AGACGACCAGAACCCTCACT | 142 | This study |
|  |  | R.398 | TGCTTTATGGGCCAACCAGT |  |  |
| *MTOR* | NM_001285748.1 | F.1759 | ATCACCCTTGCTCTCCGAAC | 122 | This study |
|  |  | R.1880 | GCCTCCATACGGATCTCCTTG |  |  |
| *NUPR1* | XM_005697656.3 | F. 318 | AGACATTATGACCACCTTCCC | 149 | This study |
|  |  | R. 466 | TCTTGCTGCGACCTTTCC |  |  |
| *PRKAA1* | XM_018065500.1 | F.4 | ATGCGCAGACTCAGTTCCTG | 197 | This study |
|  |  | R.200 | AGGCTTCGAATCTTCTGCCG |  |  |
| *RPS6KB1* | NM_001285641.1 | F.1328 | GAACACCTGTCAGCCCAGTC | 193 | This study |
|  |  | R.1501 | TTGTACGGTCCGGAGTTTGG |  |  |
| *MRPL39* | XM_005674737.1 | F. 370 | AGGTTCTCTTTTGTTGGCATCC | 101 | (1) |
|  |  | R. 470 | TTGGTCAGAGCCCCAGAAGT |  |  |
| *RPS9* | XM_005709411.1 | F.72 | CCTCGACCAAGAGCTGAAG | 64 | (1) |
|  |  | R.135 | CCTCCAGACCTCACGTTTGTTC |  |  |
| *UXT* | XM_005700842.1 | F. 270 | TGTGGCCCTTGGATATGGTT | 101 | (1) |
|  |  | R.370 | GGTTGTCGCTGAGCTCTGTG |  |  |

Supplementary Table S3. Antibodies used in the signaling protein analysis

| Name*^1^* | Company | Catalog Number |
| --- | --- | --- |
| Mouse Anti-AMPK^1^ | Meibiao Biotechnology | HZ56123 |
| Mouse Anti-mTOR^2^ | Meibiao Biotechnology | HZ99107 |
| Mouse Anti-p-AMPK^1^ | Meibiao Biotechnology | HZ56126 |
| Mouse Anti-p-mTOR^2^ | Meibiao Biotechnology | HZ99102 |
| Rabbit anti-mouse IgG H&L (HRP） | Meibiao Biotechnology | HZ7075 |

*^1^* AMPK: the AMP-activated protein kinase; mTOR: mammalian target of rapamycin

Supplementary Table S4. Effects of increasing mammary glucose supply by external pudendal artery on composition and milk protein yield.

| Items | Infused glucose, g/d | | | | | | SEM | *P*-value | | |
| --- | --- | --- | --- | --- | --- | --- | --- | --- | --- | --- |
|  | 0 | 20 | 40 | 60 | 80 | 100 |  | Linear | Quadratic | Cubic |
| Milk protein yield, g/d | 27.0 | 28.4 | 30.4 | 33.0 | 28.0 | 30.7 | 1.51 | 0.12 | 0.13 | 0.09 |
| Milk protein content, % | 3.88 | 3.79 | 3.76 | 3.75 | 3.61 | 4.07 | 0.17 | 0.78 | 0.14 | 0.24 |

Supplementary Table S5. Effects of increasing mammary gland glucose supply through the external pudendal artery on AA concentration in external pudendal artery of lactating dairy goats.

| Item*^1^* | Infused glucose, g/d | | | | | | SEM | *P*-value | | |
| --- | --- | --- | --- | --- | --- | --- | --- | --- | --- | --- |
|  | 0 | 20 | 40 | 60 | 80 | 100 |  | Linear | Quadratic | Cubic |
| Essential AA, μmol/L |  |  |  |  |  |  |  |  |  |  |
| Arg | 127 | 126 | 145 | 161 | 134 | 104 | 17.1 | 0.66 | 0.09 | 0.74 |
| His | 69.0 | 61.8 | 81.7 | 99.6 | 103 | 64.5 | 10.4 | 0.24 | 0.19 | 0.28 |
| Ile | 96.2 | 91.1 | 117 | 123 | 123 | 81.8 | 14.2 | 0.11 | 0.27 | 0.96 |
| Leu | 101 | 129 | 166 | 180 | 172 | 108 | 25.6 | 0.45 | 0.06 | 0.44 |
| Lys | 64.7 | 65.1 | 69.4 | 70.4 | 52.7 | 49.3 | 5.48 | 0.11 | <0.01 | 0.35 |
| Met | 19.6 | 24.3 | 31.8 | 36.1 | 30.8 | 20.3 | 3.42 | 0.33 | 0.01 | 0.43 |
| Phe | 53.6 | 58.3 | 68.3 | 82.9 | 69.3 | 51.3 | 5.38 | 0.21 | 0.57 | 0.55 |
| Thr | 130 | 146 | 154 | 187 | 144 | 133 | 17.3 | 0.55 | 0.08 | 0.23 |
| Val | 190 | 208 | 217 | 270 | 243 | 162 | 16.2 | 0.48 | 0.01 | 0.14 |
| Non-essential AA, μmol/L |  |  |  |  |  |  |  |  |  |  |
| Ala | 165 | 150 | 205 | 241 | 246 | 161 | 26.1 | 0.38 | 0.37 | 0.42 |
| Asx | 23.3 | 17.4 | 24.6 | 32.7 | 38.0 | 25.2 | 4.18 | 0.24 | 0.17 | 0.33 |
| Cys | 64.3 | 77.8 | 87.9 | 95.8 | 75.6 | 68.1 | 12.9 | 0.25 | 0.13 | 0.41 |
| Glx | 186 | 170 | 210 | 226 | 208 | 169 | 10.3 | 0.64 | 0.04 | 0.39 |
| Gly | 178 | 199 | 220 | 186 | 181 | 162 | 23.3 | 0.47 | 0.21 | 0.65 |
| Pro | 97.4 | 96.0 | 129 | 166 | 198 | 98.9 | 34.1 | 0.87 | 0.13 | 0.21 |
| Ser | 66.6 | 73.8 | 91.3 | 106 | 92.0 | 59.2 | 17.3 | 0.32 | 0.14 | 0.70 |
| Tyr | 63.1 | 63.9 | 81.8 | 84.4 | 75.0 | 53.5 | 11.4 | 0.28 | 0.13 | 0.55 |
| Essential AA, μmol/L | 850 | 910 | 1050 | 1211 | 1073 | 774 | 162 | 0.68 | 0.11 | 0.43 |
| Non-essential AA, μmol/L | 843 | 848 | 1048 | 1138 | 1113 | 797 | 122 | 0.56 | 0.13 | 0.73 |
| Group-1 AA, μmol/L | 205 | 208 | 264 | 303 | 279 | 190 | 39.2 | 0.62 | 0.16 | 0.88 |
| Group-2 AA, μmol/L | 708 | 766 | 869 | 992 | 870 | 638 | 128 | 0.44 | 0.13 | 0.98 |
| Branched-chain AA, μmol/L | 386 | 428 | 500 | 574 | 539 | 352 | 78.3 | 0.42 | 0.13 | 0.83 |
| Total AA, μmol/L | 1693 | 1759 | 2098 | 2349 | 2186 | 1571 | 284 | 0.64 | 0.11 | 0.48 |

*^1^* Group-1 AA: His, Met, Phe, and Tyr; Group-2 AA: Arg, Ile, Leu, Lys, Thr, and Val; Branched-chain AA: Ile, Leu, and Val. Glx: glutamate + glutamine. Asx: aspartate + asparagine.

Supplementary Table S6. Effects of increasing the mammary glucose supply on the AA supply to the mammary gland of lactating dairy goats ^1^.

| Item *^2^* | Infused glucose, g/d | | | | | | SEM | *P*-value | | |
| --- | --- | --- | --- | --- | --- | --- | --- | --- | --- | --- |
|  | 0 | 20 | 40 | 60 | 80 | 100 |  | Linear | Quadratic | Cubic |
| Essential AA, mmol/d |  |  |  |  |  |  |  |  |  |  |
| Arg | 44.4 | 46.0 | 61.0 | 72.6 | 57.7 | 44.1 | 5.72 | 0.36 | <0.01 | 0.19 |
| His | 24.2 | 22.6 | 34.3 | 44.8 | 44.5 | 27.4 | 7.14 | 0.14 | 0.07 | 0.12 |
| Ile | 33.7 | 33.3 | 49.3 | 55.4 | 52.8 | 34.8 | 9.77 | 0.37 | 0.06 | 0.23 |
| Leu | 35.1 | 47.2 | 69.7 | 81.2 | 74.3 | 46.0 | 21.7 | 0.38 | 0.09 | 0.28 |
| Lys | 22.7 | 23.8 | 29.1 | 31.7 | 22.6 | 21.0 | 3.12 | 0.20 | <0.01 | 0.25 |
| Met | 6.87 | 8.87 | 13.4 | 16.3 | 13.3 | 8.61 | 2.21 | 0.17 | <0.01 | 0.26 |
| Phe | 18.8 | 21.3 | 28.7 | 37.3 | 29.8 | 21.8 | 6.32 | 0.38 | 0.07 | 0.38 |
| Thr | 45.5 | 53.4 | 64.5 | 84.2 | 61.8 | 56.3 | 6.62 | 0.14 | <0.01 | 0.12 |
| Val | 66.6 | 75.8 | 91.2 | 122 | 105 | 68.9 | 15.8 | 0.33 | 0.02 | 0.18 |
| Non-essential AA, mmol/d |  |  |  |  |  |  |  |  |  |  |
| Ala | 57.8 | 54.8 | 86.1 | 109 | 106 | 68.5 | 16.1 | 0.13 | 0.06 | 0.12 |
| Asx | 8.17 | 6.33 | 10.3 | 14.7 | 16.3 | 10.7 | 3.35 | 0.13 | 0.36 | 0.11 |
| Cys | 22.5 | 28.4 | 36.9 | 43.1 | 32.5 | 28.9 | 7.83 | 0.20 | 0.06 | 0.19 |
| Glx | 65.5 | 62.2 | 88.0 | 102 | 89.3 | 72.0 | 11.2 | 0.13 | 0.03 | 0.14 |
| Gly | 62.3 | 72.6 | 92.2 | 83.8 | 77.8 | 68.7 | 12.6 | 0.69 | 0.09 | 0.76 |
| Pro | 34.1 | 35.0 | 54.0 | 74.7 | 85.2 | 42.1 | 17.8 | 0.29 | 0.16 | 0.37 |
| Ser | 23.3 | 26.9 | 38.3 | 47.7 | 39.6 | 25.2 | 6.12 | 0.38 | <0.01 | 0.23 |
| Tyr | 22.1 | 23.3 | 34.3 | 38.0 | 32.3 | 22.8 | 5.08 | 0.45 | 0.02 | 0.23 |
| Essential AA, mmol/d | 298 | 332 | 441 | 545 | 461 | 329 | 73.5 | 0.37 | <0.01 | 0.30 |
| Non-essential AA, mmol/d | 295 | 310 | 440 | 512 | 478 | 339 | 63.1 | 0.39 | 0.02 | 0.27 |
| Group-1 AA, mmol/d | 71.9 | 76.0 | 111 | 136 | 120 | 80.6 | 12.1 | 0.24 | 0.02 | 0.16 |
| Group-2 AA, mmol/d | 248 | 279 | 365 | 447 | 374 | 271 | 49.7 | 0.39 | <0.01 | 0.30 |
| Branched-chain AA, mmol/d | 135 | 156 | 210 | 258 | 232 | 150 | 33.1 | 0.46 | <0.01 | 0.33 |
| Total AA, mmol/d | 593 | 642 | 881 | 1057 | 940 | 668 | 154 | 0.53 | 0.02 | 0.41 |

^1^ AA supply (mmol/d) = Arterial AA concentration (mmol/L) × Mammary gland plasma flow (L/d).

^2^ Glx: glutamate + glutamine; Asx: aspartate + asparagines; Group-1 AA: His, Met, Phe, and Tyr; Group-2 AA: Arg, Ile, Leu, Lys, Thr, and Val; Branched-chain AA: Ile, Leu, and Val.

Supplementary Table S7. Effects of increasing mammary gland glucose supply through external pudic artery on mammary clearance rate of amino acids (AA).

| Item *^1^* | Infused glucose, g | | | | | | SEM | *P*-value | | |
| --- | --- | --- | --- | --- | --- | --- | --- | --- | --- | --- |
|  | 0 | 20 | 40 | 60 | 80 | 100 |  | Linear | Quadratic | Cubic |
| Essential AA, L/h |  |  |  |  |  |  |  |  |  |  |
| Arg | 3.96 | 2.92 | 6.09 | 11.6 | 5.38 | 4.15 | 2.97 | 0.58 | 0.17 | 0.24 |
| His | 11.6 | 7.57 | 10.3 | 14.0 | 13.4 | 12.7 | 3.96 | 0.08 | 0.97 | 0.31 |
| Ile | 16.3 | 13.6 | 20.2 | 29.7 | 26.4 | 23.6 | 5.42 | 0.08 | 0.43 | 0.17 |
| Leu | 29.3 | 22.9 | 27.6 | 26.6 | 33.7 | 32.6 | 4.01 | 0.17 | 0.34 | 0.27 |
| Lys | 6.36 | 7.66 | 14.4 | 15.2 | 8.10 | 5.24 | 2.20 | 0.84 | <0.01 | 0.32 |
| Met | 14.6 | 18.7 | 32.9 | 22.1 | 22.7 | 19.4 | 5.38 | 0.57 | 0.07 | 0.68 |
| Phe | 6.77 | 9.30 | 9.81 | 8.71 | 6.39 | 13.9 | 1.79 | 0.09 | 0.40 | 0.26 |
| Thr | 4.85 | 16.1 | 9.14 | 14.3 | 10.0 | 7.84 | 3.33 | 0.94 | 0.09 | 0.37 |
| Val | 23.8 | 32.6 | 29.6 | 28.6 | 27.7 | 36.0 | 7.14 | 0.46 | 0.94 | 0.54 |
| Non-essential AA, L/h |  |  |  |  |  |  |  |  |  |  |
| Ala | 6.25 | 4.19 | 8.04 | 8.13 | 4.45 | 9.15 | 2.61 | 0.56 | 0.90 | 0.47 |
| Asx | 30.0 | 11.5 | 36.0 | 31.9 | 30.0 | 15.5 | 6.32 | 0.70 | 0.13 | 0.48 |
| Cys | 2.31 | 3.28 | 5.11 | 5.65 | 4.47 | 3.57 | 0.87 | 0.15 | <0.01 | 0.12 |
| Glx | 4.90 | 4.84 | 15.7 | 16.3 | 7.04 | 8.61 | 3.12 | 0.32 | 0.02 | 0.08 |
| Gly | 7.39 | 4.02 | 17.2 | 17.1 | 7.14 | 9.57 | 3.50 | 0.51 | 0.06 | 0.21 |
| Pro | 2.06 | 5.45 | 11.7 | 1.39 | 10.1 | 3.85 | 9.38 | 0.87 | 0.65 | 0.84 |
| Ser | 27.2 | 22.7 | 31.0 | 31.2 | 16.4 | 18.4 | 4.73 | 0.11 | 0.15 | 0.14 |
| Tyr | 23.4 | 29.4 | 21.7 | 27.5 | 34.9 | 40.2 | 6.81 | 0.06 | 0.34 | 0.28 |
| Essential AA, L/h | 118 | 131 | 160 | 171 | 154 | 155 | 20.2 | 0.11 | 0.18 | 0.12 |
| Non-essential AA, L/h | 101 | 82.0 | 141 | 133 | 110 | 105 | 21.6 | 0.56 | 0.17 | 0.17 |
| Group-1 AA, L/h | 56.4 | 64.9 | 74.7 | 72.3 | 77.4 | 86.1 | 11.4 | 0.07 | 0.86 | 0.08 |
| Group-2 AA, L/h | 84.5 | 95.8 | 107 | 126 | 111 | 109 | 12.8 | 0.09 | 0.16 | 0.32 |
| Branched-chain AA, L/h | 69.3 | 69.1 | 77.4 | 85.0 | 87.8 | 92.1 | 11.7 | 0.09 | 0.99 | 0.54 |
| Total AA, L/h | 219 | 213 | 301 | 304 | 264 | 261 | 32.1 | 0.18 | 0.09 | 0.14 |

*^1^* Group-1 AA: His, Met, Phe, and Tyr; Group-2 AA: Arg, Ile, Leu, Lys, Thr, and Val; Branched-chain AA: Ile, Leu, and Val. Glx: glutamate + glutamine. Asx: aspartate + asparagine.


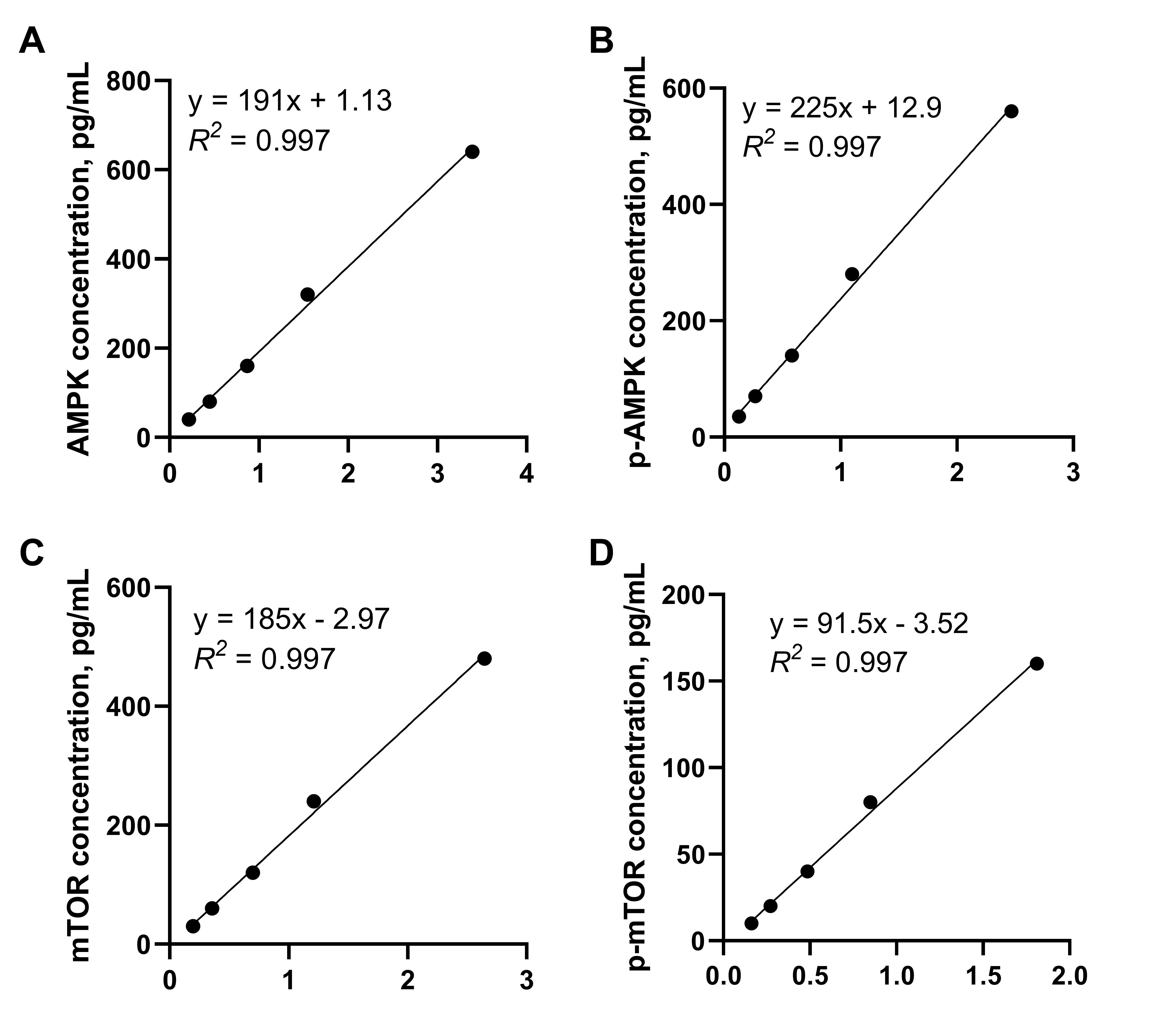
Supplementary Figure S1. Standard curve for the determination of signaling proteins.

**REFERENCES**

1. Bionaz, M., and J. J. Loor. 2007. Identification of reference genes for quantitative real-time PCR in the bovine mammary gland during the lactation cycle. Physiol. Genomics. 29:312-319.
